# miR-28: A Tiny Player in Cancer Progression and Other Human Diseases

**DOI:** 10.3390/biom15060757

**Published:** 2025-05-24

**Authors:** Karol Kotarski, Marta Kot, Klaudia Skrzypek

**Affiliations:** Department of Transplantation, Institute of Pediatrics, Faculty of Medicine, Jagiellonian University Medical College, 30-663 Krakow, Poland; k.kotarski@uj.edu.pl

**Keywords:** miR-28, miRNA, cancer, oncomiR, oncogene, metastasis, biomarker, non-coding RNA, circRNA, lncRNA

## Abstract

MicroRNAs belong to a class of small non-coding RNA molecules that regulate gene expression post-transcriptionally. By binding to specific mRNA sequences, microRNAs can either inhibit translation or promote transcript degradation. MicroRNA-28 (miR-28) plays a pivotal role in regulating the processes responsible for the pathogenesis of numerous diseases. Its function is contingent upon the specific type of disease and the cellular microenvironment. miR-28 can act as both an inhibitor and inducer of pathogenic processes. This article discusses the impact of miR-28 on the progression of various types of cancer, with particular emphasis on its role as a regulator of gene expression involved in cell proliferation, apoptosis, invasion, migration, and metastasis. Additionally, the article delves into the role of miR-28 in other human diseases and its influence on the processes that underlie their development. A comprehensive understanding of the precise mechanisms through which this specific microRNA exerts its regulatory functions could significantly impact the development of novel therapies. Furthermore, there is potential for miR-28 to be utilized as a diagnostic and preventative biomarker.

## 1. Introduction

In 1993, Victor Ambros and Gary Ruvkun first described microRNAs and their functions in the nematode *C. elegans*, thereby initiating intense research on the role these molecules play in living organisms. In their studies, they demonstrated that the *lin-4* gene encodes small RNA molecules with sequences that are complementary to the 3′ UTR regions in the mRNA of the *lin-14* gene, thus, regulating the translation of this gene’s transcription through RNA-RNA interaction [1,2]. In 2024, they were awarded the Nobel Prize in Physiology or Medicine for their scientific discoveries.

MicroRNAs are small non-coding RNA molecules 20–24 nucleotides in length known for their ability to post-transcriptionally regulate gene expression. MicroRNA molecules can bind to mRNAs, leading to transcript degradation or translation inhibition [3,4]. Due to the tremendous impact of miRNAs on protein regulation, they are crucial for maintaining body homeostasis. Previous studies have shown that their dysregulated levels can cause many dangerous diseases. Among other things, it has been discovered that dysregulated miRNAs are associated with the occurrence of numerous heart diseases [5], as well as autoimmune diseases such as systemic lupus erythematosus and rheumatoid arthritis [6]. However, special attention is paid to the impact of miRNAs on the development and progression of various types of cancer, which, due to their higher incidence and high mortality rate, pose a serious challenge to the health care system [7].

MicroRNAs are responsible for many cellular processes such as differentiation, proliferation, growth, and development [8]. Therefore, these molecules are being studied for their role in tumorigenesis. One of the miRNAs implicated in cancer development is miR-28. Its role in tumorigenesis has been described by many research groups, and this article reviews the current literature on this topic. We also investigate the function of miR-28 in other non-oncological diseases, including immunological and neurodegenerative diseases. Understanding miRNA function requires insight into the molecular steps governing their biogenesis.

## 2. MicroRNA Biogenesis

**Figure 1:**
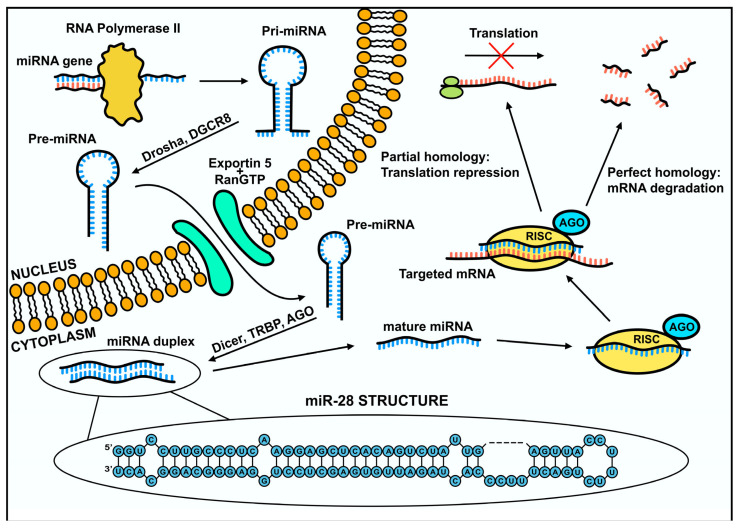
Scheme of miRNA biogenesis and its role in gene expression regulation. miRNA is transcribed from DNA by RNA polymerase II into primary miRNA (pri-miRNA). The pri-miRNA is then processed by the microprocessor complex, including Drosha, into precursor miRNAs (pre-miRNAs). Pre-miRNAs are subsequently exported from the nucleus to the cytoplasm via Exportin-5. In the cytoplasm, the pre-miRNA is cleaved by the Dicer enzyme, generating a double-stranded miRNA. The miRNA duplex binds to the RNA-induced silencing complex (RISC), where the Argonaute protein is the active part necessary for preventing protein production. The mature strand remains in RISC and binds to target mRNA for gene regulation, while the passenger strand is degraded. Depending on the degree of complementarity, miRNA regulates gene expression through mRNA target cleavage, translational repression, or mRNA degradation.

The synthesis of miRNA begins with the transcription of miRNA genes by RNA polymerase II, which forms molecules that are approximately 80 nucleotides long, containing a 3′ poly(A) tail and a 5′ cap [9]. This structure is a primary transcript called pri-miRNA. These transcripts are then recognized and cleaved by a nuclear ribonuclease, Drosha RNase III, and then fused to the DiGeorge syndrome critical region 8 (DGCR8) protein, which together form a microprocessor complex. The Drosha ribonuclease cleaves hairpin fragments from the pri-miRNA to form pre-miRNA approximately 60 nucleotides in length [4,10,11]. Then, the pre-miRNA is transported from the cell nucleus into the cytoplasm via exportin-5/RanGTP [11,12]. Once the pre-miRNA is exported to the cytoplasm, it undergoes further processing by the RNase III endonuclease Dicer. After this step, a miRNA duplex is formed, and each strand has a target length of about 20–24 nucleotides. Subsequently, the duplex containing the 5p and 3p strands is cleaved into two separate strands. The 5p strand is then usually incorporated into the miRISC (miRNA-induced silencing complex), and the 3p strand may be degraded or perform regulatory functions in the cell as well (Figure 1) [11,13]. Once mature, miRNAs are incorporated into the RISC, they become functionally competent to regulate gene expression at the post-transcriptional level.

## 3. Mechanism of Gene Expression Regulation via miR-28

The fully formed miRISC regulates protein levels in the cell [4]. The AGO (argonaute) family proteins, which bind to mature miRNAs, play an important role in this complex [14]. The microRNAs incorporated into RISC are used to recognize the complementary sequences in the 3′UTR region of the target mRNA [15]. The type of regulation depends on the complementarity of the target sequence with the miRNA and the type of AGO protein incorporated into the complex. Typically, when the sequence is highly complementary, it results in mRNA degradation. When the nucleotide match is lower, inhibition of transcript translation occurs (Figure 1) [3]. The mechanism of transcript degradation is initiated by the attachment of miRISC to mRNA, which activates binding of PABP (Poly(A)-Binding Protein), PAN2-PAN3 (poly(A)-nuclease, poly(A) nuclease), and CCR4-NOT (carbon catabolite repression- negative on TATA) complexes, which are each responsible for the process of poly(A) deadenylation. Subsequently, the DCP2 (decapping protein 2) protein, with associated cofactors, removes the cap from the 5′ end of the transcript. Deadenylation and decapping lead to transcript degradation facilitated by 5′ to 3′ exoribonuclease 1 (XRN1) [14].

In mammalian cells, translation inhibition is responsible for 6–26% of all gene expression regulatory processes [14]. Various possible mechanisms of inhibition have been proposed. One of the possibilities is regulation through the modification of the cap-dependent translation process and the poly(A) tail. Transcript translation depends on the eIF4F complex that binds the 5′ cap of mRNA. The complex consists of three subunits. One of these subunits is the eIF4G factor, which interacts with the PABP protein. The disruption of PABP binding and its interaction with eIF4G, as well as deadenylation of the poly(A) tail in mRNA, results in translation inhibition [16]. Transcripts that are regulated are then translocated to specific sites in the cell called P-bodies (processing bodies), where they can be stored or degraded [17]. Understanding the molecular mechanisms underlying miR-28 activity provides a foundation for further investigation into its role in pathological processes, particularly in tumorigenesis.

## 4. Significance of miR-28 in Cancer Progression

### 4.1. Mechanisms of Cancer Progression

The process of tumorigenesis is often associated with exposure to chemical agents, ionizing and UV radiation, and the result of inherited predispositions [18,19,20]. Nevertheless, it may also be a consequence of unhealthy lifestyles, including smoking, alcohol overconsumption, poor diet, or physical inactivity [18,19].

The tumorigenic process is initiated by DNA damage, which consequently leads to the transformation of normal cells into cancer cells with increased survival, migration, and invasion capabilities. Carcinogenesis also involves the formation of the tumor microenvironment through remodeling the extracellular matrix (ECM) [21,22]. Tumor cells proliferate rapidly, leading to intense tumor growth. At the same time, cells acquire the ability to evade the immune system’s response by losing the MHC I antigen presentation mechanism. This allows tumor cells to avoid recognition by CD8 T cells [23]. Furthermore, angiogenesis processes are activated, which promotes disease progression [24]. In subsequent stages of progression, tumor cells can migrate and invade other tissues. This results from the epithelial–mesenchymal transition (EMT), associated with the loss of E-cadherin, which is crucial for maintaining intercellular connections [25].

### 4.2. The Dualistic Role of miRNA in Carcinogenesis

MicroRNAs play an important role in the processes of carcinogenesis. They can act as both tumor suppressors by regulating oncogenes and be responsible for inhibiting tumor suppressor genes, thereby enhancing oncogenic processes. MicroRNAs that induce tumorigenesis are called oncomiRs [26]. Some miRNAs, depending on the type of cancer, can act both as oncomiRs and tumor suppressors [26]. MicroRNAs have multiple targeted genes. Thus, they can influence a wide range of pathways. Since different cancer types have their own unique carcinogenesis mechanisms, miR-28 can act differently depending on the type of cancer [27,28]. For example, miR-125b acts as an oncomiR in many hematological malignancies, but it can also act as a tumor suppressor in a wide range of solid tumors [29]. This dualistic role may be explained by the fact that miR-125b target both anti-apoptotic factors (BCL2L2, BCL2, and MCL1), pro-apoptotic factors (MAPK14, TP53, BMF, BAK1, and BBC3), metastasis promoters (LIN28B, ARID3B, and MMP13), metastasis inhibitors (STARD13, TP53, and TP53INP1), and pro-proliferative factors (STAT3, E2F3, JUN, IL6R, and ERBB2/3) [29]. Therefore, the balance between the expression of oncogenes and tumor suppressors likely determines whether miRNA is an oncomiR or tumor suppressor in different cancer types [29]. The dysregulation of miRNAs affects key cellular processes, leading to increased proliferation, inhibition of apoptosis, and enhanced migration and invasion ability. Furthermore, miRNA dysregulation also promotes angiogenesis, which is necessary for further tumor growth [27].

**Figure 2:**
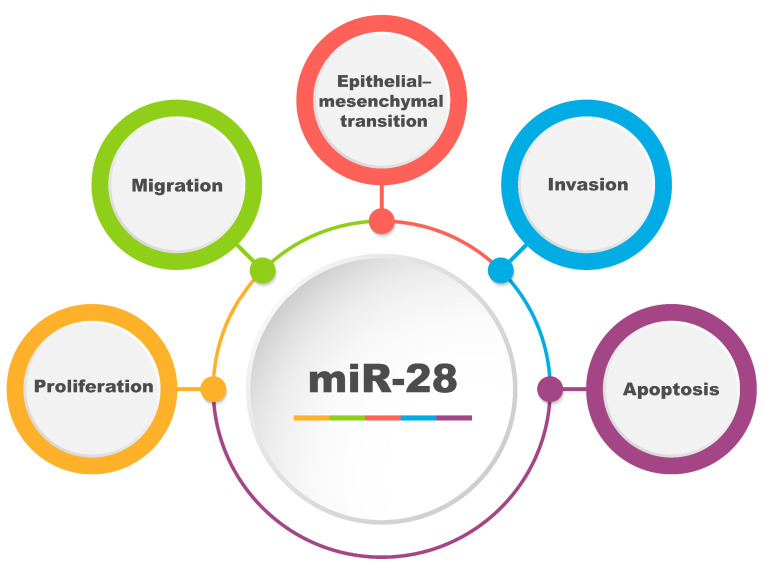
Schematic representation of key cellular processes regulated by miR-28, including proliferation (increase in cell number through division), migration (directed cell movement), epithelial–mesenchymal transition (EMT: a process in which epithelial cells acquire mesenchymal and migratory properties), invasion (the penetration of surrounding tissues), and apoptosis (programmed cell death essential for tissue homeostasis). These processes are central for determining the fate of cells and are frequently dysregulated in pathological conditions, particularly cancer. This figure highlights the regulatory role of miR-28 and its potential implications in disease progression and targeted therapeutic development.

Numerous studies have shown that both the upregulation and downregulation of miR-28 can cause changes in tumorigenesis-related processes [7]. Like other miRNAs, miR-28 can also act as both a suppressor and an oncomiR [7,30]. Therefore, comprehensive studies are being conducted to understand the role which miR-28 plays in the molecular mechanisms of tumorigenesis. Figure 2 shows the key cellular processes regulated by miR-28.

### 4.3. Regulation of miR-28 in Cancer: lncRNA and circRNA

Studies indicate that the levels of various miRNAs in cancer cells are dysregulated [31]. These alterations may be driven by several factors, including long non-coding RNAs (lncRNAs) and circular RNAs (circRNAs), which interact with miRNAs in various ways to modulate gene expression [32]. They can act as miRNA sponges, preventing them from binding to mRNAs, thus protecting transcripts from degradation. In addition, they can modulate transcription factors, enabling their binding to promoters and regulating gene expression [32].

Long non-coding RNAs (lncRNAs) belong to a class of RNA molecules that are longer than 200 nucleotides and are usually not translated into proteins. LncRNAs affect gene expression, chromatin dynamics, differentiation, and development [33,34]. Many types of lncRNAs modulate miR-28 expression in various types of cancer (Table 1). The effect of lncRNAs has been observed, e.g., in pancreatic cancer, in which the lncRNAs LOXL1-AS1 and LINC00514 caused miR-28-level disorders, resulting in the deregulation of expression of genes crucial for tumorigenesis [35,36]. A similar interaction was observed in melanoma, where lncLUADT1 and UCA1 affected tumor progression [37,38]. The influence of lncRNAs on miR-28 levels has also been reported in breast cancer, where lncRNA MCM3AP-AS1 levels were associated with increased tumor progression. A similar effect has been observed in lung cancer and many other cancer types [7,39].

Circular RNAs are a class of single-stranded RNA molecules identified in various viruses and organisms, including plants and animals. Due to their circular structure, these molecules have increased stability and are more resistant to degradation than linear RNAs [34,40]. Their function is not yet well understood. Nevertheless, numerous studies show that severe circular RNAs can affect tumorigenesis by interacting with miR-28 (Table 1). One of them is circ-CSNK1G1, which, through the suppression of miR-28-5p, causes overexpression of the LDHA gene in breast cancer, leading to increased proliferation, migration, and invasion [41]. Another example is circ-002178, which facilitates the evasion of the immune response in adenocarcinoma by translocating via exosomes to CD8^+^ T cells. The binding of miR-28-5p to circ-002178 leads to increased expression of the PD1 receptor, which inhibits the immune anti-cancer response [42]. On the other hand, circ-AHNAK is confirmed to act as an inhibitor of tumorigenesis. Its overexpression in ovarian cancer decreases the proliferation and migration of tumor cells, while increasing apoptosis and preventing EMT. The effect of circ-AHNAK has been linked to its ability to sponge miR-28, which is overexpressed in ovarian cancer and acts as an oncomiR in this tumor [43]. Other circular RNAs that affect miR-28 in cancer are circ-0001068 (ovarian cancer), circ-MYBL2, and circ-AGFG1 (NSCLC) [44,45,46].

**Table 1:** lncRNA and circRNA involvement in cancer types and associated pathways. The table summarizes the roles of lncRNAs and circRNAs in various cancer types, highlighting their association with key molecular targets and pathways. These ncRNAs play a critical role in regulating cancer progression, including processes such as tumor growth, invasion, and immune evasion, by modulating specific targets and signaling pathways.

**lncRNA**	**Cancer Type**	**Targets/Pathway**	**References**
LOXL1-AS1	Endometrial cancer	miR-28-5p, RAP1B	[47]
	Pancreatic cancer	miR-28-5p, SEMA7A/CD44/EGFR	[47]
LINC00514	Pancreatic cancer	miR-28-5p, RAP1B	[35]
LUADT1	Melanoma	miR-28-5p, RAP1B	[38]
UCA1	Melanoma	miR-28-5p, HOXB3	[37]
	Colon cancer	miR-28-5p, HOXB3	[48]
MCM3AP-AS1	Breast cancer	miR-28-5p, CENPF	[39]
CCAT1	Prostate cancer	miR-28-5p, DDX5	[49]
CDKN2B-AS1	Colorectal cancer	miR-28-5p, URGCP	[50]
NORAD	Lung cancer	miR-28-3p, E2F2	[51]
CASC9	Papillary thyroid carcinoma	miR-28-3p, BCL-2/PI3K/AKT	[52]
LINC02298	Hepatocellular Carcinoma	miR-28-5p, CCDC6	[53]
**circRNA**	**Cancer Type**	**Targets/Pathway**	**References**
circ-CSNK1G1	Triple-negative breast cancer	miR-28-5p, LDHA	[41]
circ-002178	Lung adenocarcinoma	miR-28-5p, PDL1/PD1	[42]
circ-AHNAK	Ovarian cancer	miR-28-5p, EIF2B5/JAK2/STAT3	[43]
circ-0001068	Ovarian cancer	miR-28-5p, PD1	[44]
circ-MYBL2	Non-small-cell lung cancer	miR-28-5p	[45]
circ-AGFG1	Non-small-cell lung cancer	miR-28-5p, HIF-1α	[46]

## 5. The Role of miRNA-28 in Different Types of Cancer

**Figure 3:**
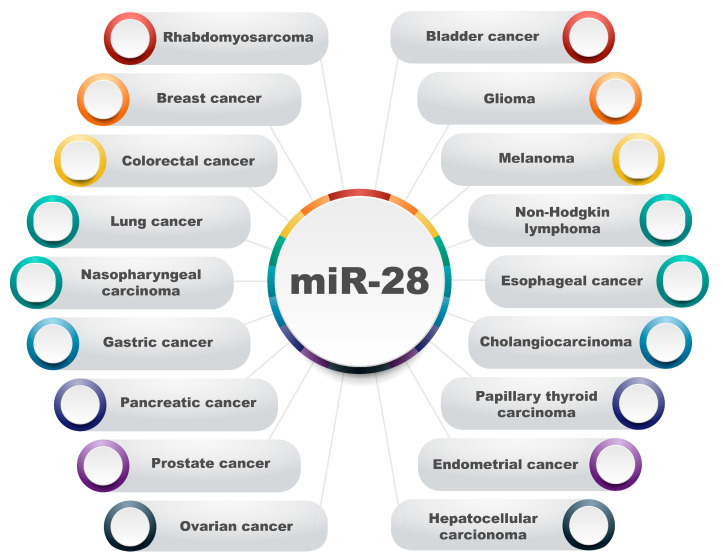
Overview of human cancer types associated with dysregulation of miR-28. The diagram presents a broad spectrum of malignancies in which the aberrant expression or functional disruption of miR-28 was implicated. These include both solid tumors and hematological malignancies, highlighting the pleiotropic nature of miR-28 across diverse tissue types. miR-28 has been associated with sarcomas such as rhabdomyosarcoma; carcinomas of epithelial origin including breast, colorectal, lung, nasopharyngeal, gastric, pancreatic, prostate, ovarian, bladder, esophageal, cholangiocarcinoma, endometrial, hepatocellular, and papillary thyroid cancers; and gliomas representing tumors of glial cell origin and melanomas derived from melanocytes. Additionally, miR-28 is implicated in hematological malignancies such as non-Hodgkin lymphoma, indicating its relevance beyond solid tumors. Dysregulation of miR-28 in these cancers involves diverse mechanisms, including altered expression levels, loss of tumor-suppressive functions, or gain of oncogenic activity, depending on the tumor context. This figure underscores the widespread involvement of miR-28 in tumor biology and supports its relevance as a potential biomarker or therapeutic target across multiple cancer types. Full references and mechanistic details are provided in the main text.

MicroRNA-28 plays an extremely important role in the development of various cancers (Figure 3). Dysregulation in this miRNA’s level has been demonstrated to induce tumorigenesis and play an active role in cancer progression by affecting the expression of genes related to oncogenesis. This section describes the impact of miR-28 on the progression of various malignancies.

### 5.1. Nasopharyngeal Carcinoma (NPC)

Nasopharyngeal carcinoma (NPC) is a type of epithelial carcinoma that originates from the mucosal epithelium of the nasopharynx. It has been observed that this cancer occurs most frequently in Southeast Asian populations, with more than 70% of new cases being diagnosed in this region [54]. Anatomy-based staging classification is not sufficient to assess prognosis, so the use of molecular biomarkers, including microRNAs, is essential [54].

Reduced levels of miR-28-5p and miR-28-3p have been reported in this rare type of malignant tumor. Significant differences between the function of strands -3p and -5p of this miRNA have been observed [55]. In nasopharyngeal cancer, miR-28-5p plays an important role in cell cycle arrest, proliferation, and cell apoptosis in vitro. MicroRNA-28-5p affects CCND1 expression, with downregulation linked to the development and progression of NPC [54]. CCND1 encodes the cyclin D1 protein, which is essential for the transition from G1 to S phase in the cell cycle. Abnormal expression can result in uncontrolled tumor growth [55,56]. The same study also reported that miR-28-3p increases the ability of NPC cells to migrate and invade [55]. The increase in migratory potential has been correlated with miR-28-3p’s effect on downregulating the expression of NM23-H1 and E-cadherin, which are crucial for epithelial–mesenchymal transition [55,57].

### 5.2. Esophageal Cancer (EC)

Esophageal cancer is most common in East Asia, East and Southern Africa, and Southern Europe. This indicates that the incidence is usually a combination of both genetic and ethnic factors, as well as lifestyle [58]. To date, the only known precursor to this disease is Barrett’s esophagus, which increases the risk of esophageal cancer by up to 40 times [58].

Due to the difficult course of the disease and the unsatisfactory results of existing therapies, new diagnostic and therapeutic methods are being tested. Thus, research groups have investigated the role of miRNAs in the pathogenesis of this cancer. Previous results suggest that miR-28-5p may be involved in the progression of esophageal cancer. It has been shown that miR-28-5p can promote tumor progression by increasing proliferation and inhibiting apoptosis via the suppression of MTSS1 (Metastasis Suppressor 1), which acts as a tumor suppressor [59].

### 5.3. Gastric Cancer (GCa)

Gastric cancer (GCa) is a malignant neoplasm with a high mortality rate, most commonly diagnosed in male patients and elderly individuals. Risk factors include smoking, overconsumption of salty and smoked foods, and insufficient consumption of fruits and vegetables [60,61]. Despite the high mortality rate, early detection of this disease significantly increases the chances of successful therapy. Therefore, the search for molecular markers that would enable more accurate diagnosis is of paramount importance. A promising candidate for further study is miR-28, which may be involved in gastric cancer tumorigenesis.

Studies of miR-28-5p expression in GCa cells show reduced levels of this miRNA compared to normal cells. Moreover, patients with higher levels of miR-28-5p had a better prognosis [61]. Expression of miR-28-5p in GCa correlates with the depth of tumor infiltration and lymph node metastasis. GCa cells with overexpression of miR-28-5p show reduced migration and invasion capacity, which consequently limits their ability to metastasize [61]. The effect of miR-28-5p on cancer cell migration is associated with inhibition of serine/threonine protein kinase (AKT) phosphorylation, which is involved in the regulation of epithelial–mesenchymal transition (EMT) [61]. Inhibition of the AKT signaling pathway suppresses EMT and cell migration [61,62]. The results suggest that miR-28-5p may inhibit gastric cancer cell migration by affecting the AKT pathway.

Increased levels of the transcription factor NRF2 have been noted in GCa, which correlates with poorer patient prognosis. Therefore, the effect of miR-28-5p on NRF2 levels has been investigated. Studies have proved that elevated levels of miR-28-5p lead to decreased NRF2 expression, limiting the ability of tumor cells to migrate [63].

Studies indicate that the deregulation of miR-28 levels in gastric cancer may also induce oncogenic effects. This is related to the regulation of the PTEN gene, where decreased expression, caused by elevated miR-28-5p levels, leads to the increased proliferation and invasiveness of tumor cells [64].

### 5.4. Colorectal Cancer (CRC)

Colorectal cancer (CRC) accounts for about 10% of all human cancer cases and is currently the fourth deadliest cancer. There are about 900,000 CRC-related deaths worldwide each year. It ranks as the second most frequently diagnosed cancer among women and the third most frequently diagnosed among men. Modern eating habits, physical inactivity, obesity, and smoking are major risk factors for CRC. The incidence of colorectal cancer is expected to increase in the coming years [65].

In colorectal cancer, miR-28 regulates the expression of the SSRP1 gene, where functions include transcriptional control and DNA damage repair [66]. Like other cancers, overexpression of SSRP1 has been reported in CRC, and high gene expression correlates with faster disease recurrence [67]. Furthermore, SSRP1 has been shown to promote tumor cell proliferation and metastasis, and affect the epithelial–mesenchymal transition in CRC. Silencing SSRP1 expression in CRC increases the efficacy of treatment with 5-fluorouracil and cisplatin [67].

MicroRNA-28-5p is a negative regulator of SSRP1. The level of miR-28-5p is often downregulated in CRC cells, and transfection with miRNA mimic sequences leads to a decrease in SSRP1 expression, resulting in reduced proliferation, migration, and invasiveness of cancer cells [67]. A similar mechanism has been observed for CAMTA2, where expression is regulated by miR-28-5p, thereby limiting CRC progression through the Wnt/β-catenin pathway [68]. MicroRNA-28-5p downregulates CCND1 (cyclin D1), a cell cycle regulatory protein considered a proto-oncogene, where overexpression is characteristic of cancer cells [56,69]. Moreover, miR-28-5p decreases levels of HOXB3, a member of the HOX protein family that plays a crucial role in cancer initiation and progression [70]. Therefore, it can be concluded that miR-28-5p contributes to reducing colorectal cancer progression by downregulating HOXB3 [69,70].

In contrast, miR-28-3p may exert opposing effects. Its increased expression significantly enhances the migration and invasiveness of CRC cells [69]. These differences result from the strand-specific effects on different genes. The opposing effect of miR-28-3p in CRC may be due to its effect on the *NM23-H1* gene [69]. The NM23-H1 protein exhibits antimetastatic properties, including inhibiting the migration and proliferation of tumor cells [71]. In colorectal cancer, miR-28-3p downregulates *NM23-H1*, resulting in an increased ability of CRC cells to migrate and invade [69].

The effect of miR-28-5p on ferroptosis has also been analyzed. Ferroptosis is a form of regulated cell death in which iron and lipid peroxides play a major role [72]. Ferroptosis can be activated by depletion of cysteine and glutathione in the cell or inhibition of glutathione peroxidase 4 (GPX4) activity. These metabolic changes result in the accumulation of reactive oxygen species (ROS), generated by a variety of mechanisms, including the Fenton reaction, which is catalyzed by iron. ROS accumulation then leads to lipid peroxidation, which is crucial for the induction of ferroptosis [72,73]. Induction of this process may provide an alternative method to eliminate treatment-resistant cancer cells. Studies show that overexpression of miR-28-5p enhances ferroptosis in colorectal cancer cells [74]. The effect of miR-28-5p on this process may be used in the future to develop novel therapeutic strategies for treating CRC.

### 5.5. Hepatocellular Carcinoma (HCC)

Hepatocellular carcinoma (HCC) may become a malignant neoplasm, and its development is associated with many factors, such as viral infections (hepatitis B and C), alcohol overconsumption, smoking, and non-alcoholic fatty liver disease [75]. The effectiveness rate of HCC treatment is high only when the disease is detected at an early stage [76].

A study of HCC revealed that hepatocellular cancer stem cells exhibit downregulated expression of miR-28-5p. Knockdown of miR-28-5p enhances tumorigenesis and promotes the self-renewal of cancer stem cells [76]. Liver cancer stem cells play a pivotal role in tumor initiation and progression, as well as tumor recurrence and resistance to treatment [76]. Overexpression of miR-28-5p in these cells inhibits tumorigenesis; thus, HCC cells became more sensitive to Sorafenib treatment. The effect of miR-28-5p in CSCs and its impact on disease progression is due to the interaction of this miRNA with insulin-like growth factor 1 (IGF-1), which can promote the development of CSCs in HCC [76].

### 5.6. Cholangiocarcinoma (CCA)

Cholangiocarcinoma is a cancer of epithelial origin, occurring more frequently in male than in female patients. The incidence rate for this type of cancer is higher in Asian and Hispanic populations and lowest among white and black populations. A particularly high incidence has been reported in the Southeast Asian region (113 cases per 100,000 population), which is associated with frequent parasitic infections caused by the hepatobiliary flukes, *Opisthorchis viverrini* and *Clonorchis sinensis* [77].

In cholangiocarcinoma, the anti-tumor effect of miR-28-5p is correlated with its effect on CD44. Increased expression of miR-28-5p results in inhibition of the cell cycle and proliferation, and reduced tumor growth in vivo. In addition, a reduced ability of cells to metastasis was observed [78].

### 5.7. Pancreatic Cancer (PC)

In recent years, the incidence of pancreatic cancer (PC) has increased significantly, and it is one of the leading causes of cancer mortality worldwide. The incidence is the highest in developed countries, especially in North America, Europe, and Australia, which is associated with increased life expectancy, an aging population, and a higher prevalence of risk factors such as obesity, diabetes, and alcohol overconsumption [79].

Studies on the effect of miR-28-5p on pancreatic cancer progression have shown that the LOXL1-AS1/miR-28-5p/SEMA7A regulatory axis is responsible for the progression of this cancer [36]. The level of miR-28-5p was found to be significantly downregulated in PC cells. Regulation of this miRNA occurs through the lncRNA LOXL1-AS1, which sponges miR-28-5p, leading to increased expression of semaphorin 7A (SEMA7A) and inducing tumor progression. Overexpression of miR-28-5p inhibits the proliferation and migration of PC cells, suggesting that it functions as a tumor suppressor in this type of cancer. Understanding the relationship between the LOXL1-AS1/miR-28-5p/SEMA7A axis may serve to develop novel therapeutic strategies [36].

It was also discovered that the downregulation of miR-28-5p in PC is affected by lncRNA LINC00514, resulting in increased expression of RAP1B. Overexpression of this gene is associated with lung metastasis and accelerates tumor growth in vivo [35].

### 5.8. Breast Cancer

In 2022, breast cancer was the most frequently diagnosed cancer among women. There has been a significant increase in the incidence of this cancer over the past four decades [80,81]. Despite the high incidence, the mechanisms underlying this disease still require detailed investigation [80].

The tumor suppressor role of miR-28 has been demonstrated in breast cancer, with studies showing that miR-28-5p may reduce cell proliferation, migration, and tumor invasion [82,41]. Analysis of the expression level of miR-28-5p in breast cancer cells showed that the level of this miRNA was significantly lower than in normal tissue. This suggests a link between miR-28-5p and tumorigenesis [82]. In addition, it has been proven that low levels of miR-28-5p are associated with lower patient survival [82]. Studies conducted on the MCF-7 breast cancer cell line have shown that increasing miR-28-5p levels by transfection leads to a decrease in their ability to migrate. On the other hand, the use of miR-28-5p inhibitors increases their migration potential [82]. In the same study, genes regulated by miR-28-5p and mechanisms of their effects on migration and tumor progression were determined using the gene chip assay. It has been shown that one of the targets of miR-28-5p is the WSB2 gene. Its dysregulated expression can induce tumorigenesis, e.g., in human melanoma cells [83]. Upregulation of WSB2 expression in breast cancer may correlate with low levels of miR-28-5p. Increased levels of this miRNA lead to downregulated expression of WSB2, which induces breast cancer cell migration. The association of miR-28-5p with WSB2 may, in the future, contribute to the development of more effective therapies for this type of cancer [82].

It has also been confirmed that miR-28 regulates the expression of NRF2 (NF-E2-related factor 2) [84]. The NRF2/KEAP1 pathway plays a pivotal role in maintaining redox homeostasis within cells, thereby exhibiting significant anti-inflammatory and anti-tumor properties [85]. In cancer, overexpression of NRF2 can promote cell proliferation and accelerate disease progression [85]. Furthermore, NRF2 inhibits apoptosis, promotes angiogenesis and renewal of cancer stem cells, and enhances resistance to chemotherapy and radiotherapy [85]. Consequently, elevated levels of this transcription factor may be associated with a poor prognosis. Its overexpression has also been reported in breast cancer compared to normal cells [84]. In contrast, miR-28 upregulation reduces NRF2 levels [84], which may inhibit tumor growth and improve the effectiveness of anti-cancer treatment.

A gene that may play an important role in breast cancer progression is CENPF, which encodes centromere protein F and is overexpressed in this type of cancer. Elevated levels of CENPF have been correlated with the increased ability of tumor cells to migrate, invade, and accelerate proliferation. Expression of this gene is regulated by miR-28-5p, and reduced levels of this miRNA prevent tumor growth restriction. Abnormal levels of miR-28-5p have been observed to be caused by overexpression of the lncRNA MCM3AP-AS1, which sequesters this microRNA and leads to further cellular dysfunction [39].

Another example of the effect of miR-28-5p on breast cancer progression is its regulation of the LDHA gene, which is overexpressed in various types of cancer and is associated with a poorer patient prognosis [86]. LDHA plays a pivotal role in the induction of tumorigenesis, and studies have shown that its overexpression in breast cancer correlates with increased cell proliferation and accelerated tumor growth [87]. Elevated levels of miR-28-5p have been shown to inhibit proliferation, reduce migration and invasion of tumor cells, and promote apoptosis. The data obtained show that, through its tumor-suppressive effect on LDHA, miR-28-5p inhibits breast cancer development [41].

### 5.9. Ovarian Cancer (OC)

Ovarian cancer is the eighth most prevalent type of cancer among women worldwide. In 2022, it accounted for 4.8% of all cancer-related deaths [81]. Despite the high mortality rate, the prognosis depends on the stage at which therapy is initiated. In stage I, the 10-year survival rate is 73–92%, while in stage III and IV it drops to 21% and less than 6%, respectively [88].

In ovarian cancer, miR-28-5p acts as an oncomiR and promotes the progression of this cancer. Its expression is significantly elevated compared to healthy tissue. Overexpression of miR-28-5p enhances the proliferation, migration, and invasion ability of cells and affects the cell cycle. Moreover, miR-28-5p inhibits apoptosis and promotes tumor growth in vivo [89]. It has also been found that miR-28-5p promotes epithelial–mesenchymal transition (EMT), further facilitating tumor progression. Furthermore, the mechanism of action of miR-28-5p in ovarian cancer has been linked to downregulation of N4BP1 protein, which is involved in protein degradation through ubiquitination [89,90].

### 5.10. Prostate Cancer (PCa)

Prostate cancer (PCa) is among the most prevalent forms of cancer diagnosed in males between the ages of 45 and 60, and it ranks second in terms of incidence among male cancer diagnoses [91]. According to global statistics, in 2022, it accounted for approximately 14.2% of all cancer diagnoses and was the fifth most common cause of cancer-related deaths among men [81].

Decreased expression of miR-28-3p was found in prostate cancer cells. Transfection of these cells with miRNA mimics led to inhibition of proliferation, while application of miR-28-3p inhibitors had the opposite effect, increasing the rate of cell division. These results confirm the involvement of miR-28-3p in regulating the proliferation of prostate cancer cells [92].

In addition, miR-28-3p is a regulator of prostate cancer cell apoptosis. Upregulation of miR-28-3p was found to lead to increased apoptosis of cancer cells, which correlated with decreased expression of anti-apoptotic BCL-2 protein and increased levels of pro-apoptotic Bax protein [92]. The use of miR-28-3p inhibitors reversed this effect by reducing cell apoptosis [92]. Moreover, it was found that miR-28-5p may also contribute to the induction of apoptosis by downregulating the E2F6 protein, known for its anti-apoptotic properties [93].

MicroRNA-28-3p also regulates the expression of the ARF6 gene. Overexpression of ARF6 is associated with an increased ability of cancer to migrate and invade. This suggests that ARF6 may play a crucial role in prostate cancer metastasis. MicroRNA-28-3p may therefore act as a tumor suppressor miRNA and represent a potential target for anti-cancer therapy [92].

The transcription factor SREBF2 is an oncogenic protein that, in prostate cancer, regulates proliferation, cell survival, and the ability of cancer cells to migrate and invade [94]. Downregulation of its expression leads to the inhibition of the progression of this cancer. SREBF2 has been identified as a direct target of miR-28-5p. Exogenous introduction of this microRNA into cells decreases SREBF2 expression at both the mRNA and protein levels, demonstrating a tumor-suppressive role for miR-28-5p in prostate cancer [94].

### 5.11. Bladder Cancer (BCa)

Bladder cancer is the most prevalent type of cancer affecting the urinary system [95]. In approximately 75% of cases, this cancer is confined to the mucosa (non-muscle invasive bladder cancer). In 25–30% of cases, the tumor invades deeper layers of the bladder wall and infiltrates the muscle membrane or forms metastases [95]. Despite advances in diagnosis and anti-cancer therapy, long-term survival rates have remained unchanged for many years [95].

Numerous types of miRNAs have been identified in bladder cancer, suggesting their pivotal roles in disease progression, and, thus, these molecules may serve as valuable targets in medical applications. Overexpression of miR-28-5p has been detected in the urine of patients with BCa, which may have important diagnostic implications. This may allow for the early non-invasive detection of cancer and rapid implementation of appropriate therapy [96,97].

### 5.12. Non-Small-Cell Lung Cancer (NSCLC)

Non-small-cell lung cancer (NSCLC) is a malignant tumor that accounts for a significant percentage of cancer-related deaths [98,99]. In the case of NSCLC, miR-28 acts as an oncomiR, as its elevated levels promote increased proliferation of tumor cells [99]. MicroRNA-28 affects the PTEN gene, a key tumor suppressor, through mechanisms dependent on and independent of the PI3K pathway [99,100]. Decreased PTEN activity leads to increased proliferation and survival of tumor cells and affects the microenvironment that promotes new tumor growth [100]. Overexpression of miR-28 in NSCLC may induce tumor cell proliferation by inhibiting PTEN expression (activating the PI3K/AKT pathway) [99].

### 5.13. Glioma

Gliomas are the most common primary brain and spinal cord tumors [101], for which no effective therapy has yet been developed [102]. Numerous studies indicate that miR-28 significantly impacts processes involved in the progression of gliomas, including the regulation of tumorigenesis-related genes.

Studies have shown that the human *TRPM7* gene negatively regulates miR-28-5p expression, which affects glioma progression [102]. Levels of miR-28-5p in glioma cells are significantly downregulated compared to normal cells. Increasing miR-28-5p expression via transfection of miRNA mimics resulted in the inhibition of proliferation and reduced the ability of glioma cells to invade. The use of miR-28-5p inhibitors had the opposite effect, confirming the involvement of this microRNA in the mechanisms of glioma progression [102]. Further analysis showed that miR-28-5p upregulates RAP1B, a protein that participates in numerous signaling pathways involved in the processes of cell proliferation, differentiation, and apoptosis [102,103]. Considering the oncogenic nature of RAP1B in glioma and the ability of miR-28-5p to downregulate this protein, it can be inferred that miR-28-5p has an important function in the molecular mechanisms of progression in this cancer [102].

Other studies indicate that miR-28-5p in glioma may also exhibit oncogenic properties. It has been shown that it can interact with the FOXO1 gene, where reduced activity promotes the formation of tumor spheres and increases the viability of tumor cells and their ability to proliferate, promoting disease progression [104].

### 5.14. Rhabdomyosarcoma (RMS)

Rhabdomyosarcoma (RMS) is one of the most common soft tissue malignancies in individuals under 20 years of age. It accounts for approximately seven percent of cancers in children and one percent in adults [105]. RMS originates from impaired differentiation of stem cells or myogenic progenitor cells [106]. It has been demonstrated that one miRNA with a significant role in the development and progression of RMS is miR-28, which is regulated by the transcription factor SNAIL [107,108].

Studies of two RMS subtypes (alveolar and embryonal) revealed that elevated levels of miR-28-3p caused a decrease in the number of S-phase cells and an increase in the G0/G1 phase, leading to the inhibition of proliferation and diminished ability for uncontrolled cell division [106]. Higher levels of miR-28-3p also significantly inhibit cell migration in the scratch assay, suggesting its effect on limiting the tumor’s ability to metastasize [106]. The effect of miR-28-3p on the expression of myogenic regulatory factors (MRFs), such as MYOG, MYOD, MEF2A, and MSTN, was also observed in various RMS subtypes [106].

### 5.15. Melanoma

Melanoma is a malignant tumor mainly affecting the skin, eyes, and mucous membranes [109]. It occurs most often in geographical regions where individuals with light skin pigmentation are exposed to excessive sun. The risk of developing the disease is much higher in people with a genetic predisposition, but it is not the most significant risk factor [110].

In melanoma, miR-28-5p can be bound by the lncRNA LUADT1, leading to increased expression of the RAP1B gene. As a result, deregulation of the expression of this oncogene promotes intense cell proliferation [38]. Understanding the relationship between the miR-28-5p/RAP1B axis and LUADT1 may help develop effective therapeutic strategies [38]. Moreover, the lncRNA UCA1/miR-28-5p/HOXB3 axis has emerged as a key regulatory pathway [37]. In this mechanism, HOXB3 functions as a miR-28-5p target gene, while UCA1, which is overexpressed in melanoma, can bind miR-28-5p. As a result, HOXB3 gene expression is increased, facilitating tumor progression [37].

A common phenomenon observed in cancer is the presence of exhausted T cells within the tumor microenvironment. These cells are characterized by the overexpression of inhibitory receptors (IRs), diminished production of effector cytokines, and reduced cytolytic activity, which prevents effective elimination of tumor cells [111,112]. According to studies, the exhausted T cells isolated from murine melanoma exhibit dysregulated levels of specific miRNAs, including miR-28 [111]. It has been indicated that miR-28 can bind to the PD-1 (programmed cell death protein 1) receptor, leading to inhibition of anti-cancer immune response [111,113]. Transfection with miR-28 mimics to upregulate this miRNA has been shown to reduce PD-1 expression [111]. The use of miR-28 to downregulate inhibitory receptor (IR) expression and prevent tumor cells from evading immune response mechanisms may represent a promising approach in anti-cancer therapies.

### 5.16. Non-Hodgkin Lymphoma (NHL)

Non-Hodgkin lymphomas (NHLs) are the most common hematological malignancy worldwide [114]. They include B-cell lymphomas such as Burkitt lymphoma (BL), diffuse large B-cell lymphoma (DLBCL), and T/NK-cell lymphomas such as T-cell acute lymphoblastic leukemia. Risk factors vary depending on the subtype of NHL [114]. The incidence of these cancers is higher among men than women and among individuals over 65 years of age. Moreover, NHLs are often associated with autoimmune diseases, viral infections, and hereditary predisposition to hematological malignancies [114].

In Burkitt lymphoma (BL) and other types of non-Hodgkin lymphomas (NHL) originating from germinal centers, decreased expression of miR-28 was found, resulting from negative regulation of this miRNA by the MYC oncogene [115,116]. Restoration of normal miR-28 expression has been shown to impair tumor cell proliferation and clonogenic properties in BL. MicroRNA-28 acts as a tumor suppressor by affecting the MAD2L1 gene expression, which is a component of the spindle checkpoint, required to inhibit proliferation induced by this miRNA. In addition, miR-28 interacts with BAG1, a BCL2-related factor that acts as an activator of the ERK signaling pathway [115].

### 5.17. Papillary Thyroid Carcinoma (PTC)

Papillary thyroid carcinoma (PTC) is the most prevalent endocrine gland tumor, accounting for approximately 85% of all thyroid cancer cases. It is classified as an indolent cancer, which is characterized by a relatively favorable prognosis, with a 10-year survival rate of 93%. However, certain subtypes of PTC have been observed to exhibit higher malignancy and lower survival rates [117].

In the tissues of patients diagnosed with PTC, elevated levels of the lncRNA CASC9 were found. CASC9 had been shown to influence the progression of this malignancy through the sequestration of miR-28-3p. Thus, downregulating this lncRNA with siRNA resulted in increased levels of miR-28-3p. The analyses demonstrated that in the examined cell lines, increasing the level of this miRNA resulted in increased apoptosis, cell sensitivity to doxorubicin, and reduced migration and proliferation. In vivo studies in mice also showed that decreased CASC9 expression reduced tumor growth. The involvement of miR-28-3p in PTC progression is due to the upregulation of BCL-2 protein, which is known for its anti-apoptotic properties [52].

### 5.18. Shared Molecular Mechanisms Regulated by miR-28 Across Cancer Types

Across the analyzed cancer types, miR-28 appears to influence a set of shared molecular mechanisms, suggesting a conserved pattern of action. The most frequently regulated pathways include epithelial–mesenchymal transition (EMT) [55,67,89], apoptosis [52,55,59,,92,94], and the PI3K/AKT signaling cascade [52,64,99]. For example, miR-28-5p inhibits EMT by upregulating E-cadherin or downregulating NM23-H1 in colorectal cancer (CRC) [69], nasopharyngeal carcinoma (NPC) [55], and ovarian cancer (OC) [89], thereby reducing cell migration and invasiveness. Apoptotic regulation by miR-28, through the modulation of BCL2, BAX, and E2F6, has been documented in prostate cancer (PCa) [92,93] and papillary thyroid carcinoma (PTC) [52], supporting its tumor-suppressive function. Furthermore, miR-28 affects the PI3K/AKT axis in gastric cancer (GCa) [64] and non-small cell lung cancer (NSCLC) [99], with downstream consequences for proliferation, survival, and metastasis. Despite differences in expression levels and molecular targets across tumor types, these mechanistic overlaps highlight the pleiotropic role of miR-28. Depending on the tissue context and molecular landscape, miR-28 can act either as a tumor suppressor or an oncomiR, modulating a limited but highly conserved set of signaling pathways. This pleiotropy not only underscores its biological relevance but also positions miR-28 as a modulator of core oncogenic pathways.

The involvement of miR-28 in the pathogenesis and progression of various cancer types discussed in this review are summarized in Table 2. Although most studies have focused on miR-28 in cancer, it may also be useful in treating and monitoring several non-oncological diseases.

## 6. MicroRNA-28 in Non-Oncological Diseases

MicroRNAs can be used not only in cancer therapy and diagnostics but also in treating and monitoring other serious non-oncological diseases. Numerous studies indicate the key role of miR-28 in regulating inflammatory processes and its involvement in the pathogenesis of autoimmune [6], neurodegenerative [118], cardiovascular [5], and metabolic diseases [119].

### 6.1. Infectious Diseases (Pathogenic)

MicroRNA-28 has been demonstrated to play a pivotal role in the immune response to a wide range of infections caused by various pathogens, including viruses [120,121] and bacteria [122]. MicroRNA-28-3p can serve as a biomarker for identifying infections with *Helicobacter pylori*, the bacterium responsible for the development of gastric ulcers. Its presence among circulating miRNAs correlates with *H. pylori* infection and may help diagnose infections with this pathogen [122].

Furthermore, studies have demonstrated that miR-28-3p plays a crucial role in the suppression of HTLV-1 (human T-lymphotropic virus type 1) and HIV-1 replication, through the binding of miR-28-3p to specific sequences present in the viral mRNA [120,121]. MicroRNA-28 is known as an anti-HIV-1 miRNA due to its involvement in the immune response against HIV-1 infection. The 3′ end of this virus’s mRNA is a molecular target for miR-28, and its expression affects the course of infection and disease progression [121].

The virus pandemic SARS-CoV-2, which began in 2019, has led to approximately 777 million infections and 7 million deaths by February 2025 [123,124]. It has been shown that miR-28-3p may also be a therapeutic target in that disease. SARS-CoV-2 uses a spike protein (S-protein) to spread, which binds to the transmembrane protein, angiotensin-converting enzyme 2 (ACE2) [125]. ACE2 receptors are present on cells of many organs, such as the heart, brain, and lungs [125]. In vitro studies have shown that in SARS-CoV-2 virus infection, miR-28-3p levels in cells treated with the virus’ S protein are lower than in control cells. Moreover, it has been established that the molecular target for this miRNA may be the metalloproteinase ADAM17, which is involved in the mechanism of virus entry into cells via ACE2 receptors [126].

### 6.2. Metabolic and Endocrine

The prevalence of lifestyle diseases has emerged as a pressing global health concern. One of them is obesity. Its impact on metabolism and the development of cardiovascular diseases is already evident in young patients [119]. Preventing obesity in childhood can significantly reduce the risk of related diseases in adulthood. Accordingly, studies revealed that circulating microRNAs, including miR-28-3p, can be used to diagnose and detect metabolic disorders in children with obesity [119].

Diabetes mellitus is a common metabolic disorder occurring worldwide. Evidence suggests that in type 2 diabetes mellitus, miRNA levels are dysregulated, including miR-28-3p [127]. This disease is also commonly associated with various complications. One of them is diabetic retinopathy (DR), which is the leading cause of vision loss among middle-aged, economically active people [128]. Nevertheless, there is still a lack of appropriate biomarkers for its early detection. Therefore, conducted studies identified miR-28-3p as one of three potential biomarkers of DR. In addition, it has been confirmed that miRNAs may be involved in pathogenesis by affecting the proliferation of human retinal microvascular endothelial cells (HREMECs) [129].

Polycystic ovary syndrome (PCOS) is a hereditary condition that affects approximately 15% of women of reproductive age worldwide. This disease leads to ovulation disorders, infertility, obesity, and type 2 diabetes [130]. Furthermore, women diagnosed with PCOS have a higher risk of developing endometrial cancer [131]. Prokineticin 1 (PROK1) is regulated by miR-28-5p and may play a role in the pathogenesis of PCOS [132]. PROK1 is associated with ovarian physiology, endometrial receptivity, and embryo implantation [132]. Studies using a rat model indicated that PROK1 in PCOS promotes proliferation, limits cell cycle inhibition, and affects the process of apoptosis. Therefore, it is hypothesized that miR-28-5p may limit the progression of PCOS, and the miR-28-5p/PROK1 axis may be a potential therapeutic target in patients with this condition [132].

An interesting example of the application of miR-28 is its use as a biomarker in the diagnosis of sarcopenia caused by Cushing’s syndrome (CS) [133]. Cushing’s syndrome is associated with hypercortisolism resulting from overactive adrenal glands, pituitary gland, or excessive secretion of adrenocorticotropic hormone (ACTH) [133]. More than one-third of patients with CS experience muscle weakness in the lower extremities and pelvic region. The symptoms result from the inhibitory effects of steroid hormones on protein synthesis, increased catabolism, and inhibition of myogenesis through effects on myogenin, a transcription factor essential for normal muscle differentiation [134]. MicroRNA-28-5p plays a crucial role in the processes of muscle development, especially in myoblast differentiation and proliferation. Plasma levels of this miRNA are elevated in patients after CS remission. With this knowledge, it is possible to diagnose patients with an increased risk of sarcopenia, so that appropriate measures can be implemented to improve their quality of life and function [133].

### 6.3. Neurological and Neurodegenerative Diseases

Dysregulated levels of miRNAs have also been observed in Parkinson’s disease (PD), the second most common neurodegenerative disease worldwide [135]. Its incidence is expected to rise in future generations, while early detection remains a major challenge in modern medicine [135]. Consequently, it is necessary to develop new diagnostic methods to detect PD more effectively. Studies have shown that miR-28-5p can act as a biomarker in the diagnosis and assessment of the disease’s progression. Analysis of the expression of this miRNA in the serum of patients may contribute to earlier diagnosis and better monitoring of the course of the disease [136].

Neuropathic pain is defined as pain originating from damage or disease of the somatosensory nervous system [137]. Research conducted on rats with chronic constriction injury (CCI) of the sciatic nerve has shown a reduction in the expression levels of miR-28-5p. In vivo analyses showed that miR-28-5p overexpression significantly reduces levels of the proinflammatory cytokines IL-6, IL-1β, and cyclooxygenase Cox-2, which are involved in the progression of neuropathic pain [138]. It has been shown that miR-28-5p can alleviate pain by downregulating the expression of the ZEB1 gene, a key regulator of neuroinflammatory conditions also involved in developing neuropathic pain. The analyses performed indicate that the miR-28-5p/ZEB1 axis may be a potential therapeutic target for the treatment of neuropathic pain [138].

### 6.4. Autoimmune and Inflammatory Diseases

Rheumatoid arthritis (RA) is an autoimmune disease that affects approximately 1% of the global population. RA is characterized by chronic inflammation of the joints, resulting in progressive joint damage. Moreover, RA can cause additional complications, including heart, kidney, lung, and nervous system damage [139]. The involvement of miRNAs in the pathogenesis of autoimmune diseases has been scientifically confirmed [6]. Studies have shown that in RA, miR-28 can regulate the expression of genes involved in the pathogenic process. Chronic inflammation leads to exhaustion of immune cells such as NK cells and T cells [140,141]. One of the mechanisms responsible for NK cell exhaustion is the overexpression of the PD-1 receptor, which leads to reduced proliferation, reduced cytokine production, and impaired cytolytic activity. MicroRNA-28 may be involved in regulating PD-1 receptor expression, suggesting its involvement in RA-related pathogenic processes [142].

### 6.5. Circulatory Diseases

Pulmonary embolism (PE) is the third most common cause of death among hospitalized patients [143]. Studies of plasma samples from PE patients indicate that miR-28-3p levels are elevated, suggesting its potential use as a non-invasive diagnostic biomarker [144].

The diverse roles of miR-28 in tumorigenesis and other non-oncological diseases have prompted extensive research into its potential clinical applications, including diagnostics and targeted therapy.

## 7. MicroRNA-28 as a Therapeutic Target and Diagnostic Biomarker

As previously discussed in earlier sections of this publication, miR-28 has emerged as a potential biomarker with the potential to facilitate the early detection of numerous cancers. In many cases, early diagnosis has been shown to significantly enhance the efficacy of therapeutic interventions. Moreover, it can also be used to assess the clinical status of patients. Previous studies have indicated that miR-28-3p and miR-28-5p can be used as biomarkers in the diagnosis of colorectal cancer and gastric cancer [145,146,147,148]. MicroRNA-28 has also been identified as one of four circulating miRNAs with diagnostic potential in diffuse large B-cell lymphoma (DLBCL). A prognostic model based on miRNA levels in patients’ serum provides a simple and effective method for assessing their clinical status [149]. The model can also be used to diagnose the early stages of renal cell carcinoma, in which patients’ serum miR-28-5p levels are significantly reduced compared to healthy individuals [150]. There is also the potential to use miR-28-5p as a biomarker in the non-invasive diagnosis of bladder cancer by analyzing patients’ urine, which may facilitate screening [96,97].

The potential application of microRNAs in combination therapy with anti-cancer drugs is promising. This approach may enhance treatment efficacy while concurrently reducing the risk of treatment failure and limiting side effects. A notable example of this type of therapy is the combination of miR-28 and ibrutinib for the treatment of DLBCL [151]. Similarly, a combination of miR-28 with doxorubicin (adriamycin) enhances the efficacy of DLBCL treatment [152]. These results suggest that strategies based on combination therapy may also be effective in the treatment of other cancer types [151]. Furthermore, it has been shown that in DLBCL, cell proliferation can be decreased, and apoptosis can be induced by curcumin, which can upregulate miR-28-5p [153]. Another therapeutic approach is the use of antisense oligonucleotides (AMOs) and antagomiRs, which can inhibit the activity of mature miRNAs [154]. The use of microRNA masks, designed to interfere with the interaction between specific microRNA and their target mRNAs, also shows therapeutic potential. Equally promising is the use of miRNA mimics sequences that can compensate for miR-28 deficiencies in cancer cells [154]. Moreover, miRNA-28 as biomarkers can also be used not only in early cancer detection but also for diagnosing various non-oncological diseases, including Alzheimer’s disease, chronic periodontitis, leptospirosis, pulmonary embolism, and many other diseases [118,155,156]. MicroRNAs, such as miR-124-2, miR-151a-5p, miR-192-5p, and 122-5p, and many others, are also promising therapeutic and diagnostic targets [157,158].

## 8. Challenges, Limitations, and Future Perspectives in miRNA-Based Therapies

MicroRNAs hold potential for clinical use as biomarkers and therapeutic targets. However, there are still challenges that need to be addressed before safe and effective therapies can be developed.

MiRNA-based therapies may dysregulate normal cell metabolism and cellular pathways. Thus, there are concerns about potential off-target effects and toxicity to healthy tissues [159]. Moreover, a single miRNA targets a wide range of genes and can affect multiple pathways, leading to dangerous side effects [160].

Another critical challenge is finding a proper route of administration for miRNA-based drugs. Despite the small size of miRNAs, passive diffusion through the lipid membrane is not possible [26]. MicroRNAs are also vulnerable to RNases and are therefore easily degraded [159,26,161]. Low biological stability, poor water solubility, and problems with diffusion into the interior of the cell are the primary factors necessitating the development of a suitable drug delivery system.

A third major limitation of RNA-based therapeutics, mainly associated with the delivery system, is immunogenicity. Some delivery platforms, including viral vectors and lipid-based, polymer-based, or inorganic-based nanoparticles, may eventually trigger immune responses, resulting in hypersensitivity reactions or anaphylaxis [159,26,161]. MicroRNA modifications, e.g., 2′-O-methyl, locked nucleic acid (LNA) bases, also raise concerns about the possibility of triggering immune systems, which could lead to side effects [159,160].

The utilization of circulating microRNAs as non-invasive disease biomarkers demonstrates great potential. Nonetheless, there are also a few limitations concerning the use of miRNAs as biomarkers. The main challenge is the difficulty in establishing a reliable miRNA level profile for healthy and disease-affected individuals. These difficulties are mainly related to the factors that may influence miRNA levels, namely age, sex, and lifestyle elements, such as diet, BMI, and physical activity [162]. A further issue pertains to the formulation of a suitable protocol for the storage, extraction, and analysis of patient samples.

Recently, several clinical trials have been underway for the treatment of numerous diseases with miRNA-based therapeutics. They focus on various types of cancers, i.e., hepatocellular carcinoma, melanoma, pancreatic cancer, and non-small-cell lung cancer. Some of the miRNA drugs, including INT-1B3 or Cobomarsen, also target a wide range of advanced solid tumors [160,163]. Moreover, there are plenty of trials for non-oncological diseases and disorders, i.e., chronic hepatitis C, heart failure, keloid disorder, and Huntington disease. There is also a trial for MRG-110, a miR-92a-based drug, demonstrating the potential to accelerate the wound healing process [160,163,164]. Furthermore, some miRNA-based therapeutics are in the preclinical development stage [163]. The substantial number of clinical trials confirms the high potential of using miRNA therapeutics. More importantly, novel technologies for targeted drug delivery to cancer cells are currently under development. The utilization of antibody-coated nanoparticles, which are designed to target specific tumor antigens, is a current area of research. The combination of these nanoparticles with microRNAs and anti-cancer drugs has the potential to facilitate the delivery of therapeutic agents into target cells [161,165].

## 9. MicroRNA-28 in Stem Cells

The involvement of miR-28 in regulating the differentiation of human mesenchymal stem cells (MSCs) has also been demonstrated. MSCs isolated from bone marrow (BM-MSCs) are multipotent cells with low immunogenicity, characterized by a high capacity for proliferation and differentiation; therefore, they can be used to treat many diseases. In BM-MSCs undergoing osteogenesis, the expression of miR-28 is increased, indicating that this miRNA is involved in the osteogenic differentiation of these cells. It has been shown that miR-28 directly binds to the STAT1 mRNA, decreasing its expression and consequently leading to the overexpression of ACP and RUNX2, which in turn induce differentiation [166]. Stem cells are important tools for modern therapies, so studying microRNAs, including miR-28, may play an important role in the development of regenerative medicine.

## 10. Conclusions

MicroRNAs serve as key post-transcriptional regulators of gene expression, primarily modulating mRNA stability and translation. They participate in a wide range of biological processes, including cell proliferation, differentiation, and apoptosis. Among them, miR-28 plays a crucial regulatory role, and its dysregulation has been implicated in the pathogenesis of various diseases, including cancer. Notably, miR-28 exhibits a dualistic function, acting either as a tumor suppressor or an oncogene. This emphasizes the importance of elucidating its molecular mechanisms in disease development.

Recent studies highlight the potential of miR-28 as a biomarker for both diagnostic and prognostic purposes. MicroRNA-28 expression profiling offers a valuable tool for the early and non-invasive detection of pathological conditions. Ongoing research in the field of microRNAs further underscores the strategic relevance of miR-28 as a target for clinical translation. Moreover, the ability to pharmacologically modulate miR-28 levels presents a new approach for personalized therapies, particularly in oncology.

## Figures and Tables

**Table 2:** Overview of miRNA roles in cancer regulation. The table summarizes the roles of miR-28 across different cancer types, highlighting their regulated factors, functional role (e.g., tumor suppressor or oncogene), and associated biological functions, such as cell differentiation, cycle regulation, migration, and invasion.

Cancer Type	miRNA	Regulated Factors	Role	Function	References
Nasopharyngeal carcinoma	miR-28-5p	*CCND1*	Tumor suppressor	Cell cycle, apoptosis	[55]
	miR-28-3p	*NM23-H1*	OncomiR	EMT, migration, invasion	[55]
Esophageal cancer	miR-28-5p	*MTSS1*	OncomiR	Cancer progression, proliferation, apoptosis	[59]
Gastric cancer	miR-28-5p	*AKT*	Tumor suppressor	Migration, invasion	[61]
		*NRF2*	Tumor suppressor	Migration, invasion	[63]
	miR-28	*PTEN*	Tumor suppressor	Proliferation, invasion	[64]
Colorectal cancer	miR-28-5p	*SSRP1*	Tumor suppressor	Proliferation, migration, EMT	[67]
		*CCND1*	Tumor suppressor	Cell cycle, proliferation	[69]
		*HOXB3*	Tumor suppressor	Cancer progression	[69]
		*CAMTA2*	Tumor suppressor	Proliferation, metastasis	[68]
	miR-28-3p	*NM23-H1*	OncomiR	Migration, invasion	[69]
Hepatocellular carcinoma	miR-28-5p	*IGF-1*	Tumor suppressor	Cancer stem cell expansion	[76]
Cholangiocarcinoma	miR-28-5p	*CD44*	Tumor suppressor	Cancer progression, Cell cycle arrest, proliferation, metastasis	[78]
Pancreatic cancer	miR-28-5p	*SEMA7A*	Tumor suppressor	Proliferation, migration, and cancer progression	[36]
		*RAP1B*	Tumor suppressor	Tumor growth, metastasis	[35]
Breast cancer	miR-28-5p	*WSB2*	Tumor suppressor	Migration	[82]
		*NRF2*	Tumor suppressor	Cancer progression	[84]
		*CENPF*	Tumor suppressor	Migration, invasion, proliferation	[39]
		*LDHA*	Tumor suppressor	Proliferation, migration, apoptosis	[41]
Ovarian cancer	miR-28-5p	*N4BP1*	OncomiR	Proliferation, migration, invasion, EMT	[89]
Prostate cancer	miR-28-5p	*E2F6*	Tumor suppressor	Apoptosis	[93]
		*SREBF2*	Tumor suppressor	Proliferation, cell survival, migration, invasion	[94]
	miR-28-3p	*ARF6*	Tumor suppressor	Migration, invasion	[92]
		*BCL2*, *BAX*	Tumor suppressor	Apoptosis	[92]
Non-small-cellular lung cancer	miR-28	*PTEN*	OncomiR	Proliferation, cell survival, tumor microenvironment	[99]
Glioma	miR-28-5p	*RAP1B*	Tumor suppressor	Proliferation, migration	[102]
		*FOXO1*	OncomiR	Tumor spheres formation, proliferation, viability	[104]
Rhabdomyosarcoma	miR-28-3p	*MYOG*, *MYOD*, *MEF2A*, *MSTN*	Tumor suppressor	Cell differentiation, cell cycle arrest, migration, proliferation	[106]
Melanoma	miR-28-5p	*RAP1B*	Tumor suppressor	Proliferation	[38]
		*HOXB3*	Tumor suppressor	Cancer progression	[37]
	miR-28	*PD1*	Tumor suppressor	Inhibiting overexpression of inhibitory receptors (IR) on exhausted T-cells	[111]
Non-Hodgkin lymphoma	miR-28	*MAD2L1*	Tumor suppressor	Proliferation	[115]
		*BAG1*	Tumor suppressor	Proliferation, cell cycle	[115]
Papillary thyroid carcinoma	miR-28-3p	*BCL2*	Tumor suppressor	Apoptosis	[52]

## Data Availability

No new data were created or analyzed in this study.

## Abbreviations

ACE2	Angiotensin-converting enzyme 2
AKT	Serine/threonine—protein kinase
AMOs	Anti-miRNA antisense inhibitors
ARF6	ADP-ribosylation factor 6
BAG1	BAG family molecular chaperone regulator 1
BAX	Bcl-2-like protein 4
BM-MSC	Bone marrow mesenchymal stem cells
CCND1	Cycline D1 gene
CENPF	Centromere protein F
CircRNA	Circulating RNA
CSCs	Cancer stem cells
E2F6	E2F Transcription Factor 6
ECM	Extracellular matrix
EMT	Epithelial–Mesenchymal Transition
ERK	Extracellular-signal-regulated kinase
FOXO1	Forkhead box protein O1
GAGE12I	G antigen 12I
GC	Germinal center
HOXB3	Homeobox protein Hox-B3
hREMECs	Human retinal microvascular endothelial cells
IGF-1	Insulin-like growth factor 1
LDHA	Lactate dehydrogenase A
LncRNA	Long non-coding RNA
MAD2L1	Mitotic spindle assembly checkpoint protein MAD2A
MEF2A	Myocyte-specific enhancer factor 2A
miRNA	MicroRNA
MRF	Myogenic regulatory factors
MSTN	Myostatin
MYOD	Myoblast determination protein
MYOG	Myogenin
N4BP1	NEDD4 Binding Protein 1
NRF2	Nuclear factor erythroid 2-related factor 2
PD	Parkinson disease
PD1	Programmed cell death protein 1
PI3K	Phosphoinositide 3-kinases
PTEN	Phosphatase and tensin homolog
RAP1B	Ras-related protein Rap-1b
ROS	Reactive oxygen species
RUNX2	Runt-related transcription factor 2
SEMA7A	Semaphorin 7A
SNAIL	Zinc finger protein SNAI1
SREBF2	Sterol regulatory element-binding protein 2
SSRP1	Structure specific recognition protein 1
STAT1	Signal transducer and activator of transcription 1
TRPM7	Transient receptor potential cation channel subfamily M member 7
WSB2	WD repeat and SOCS box-containing protein 2
ZEB1	Zinc finger E-box-binding homeobox 1
